# Evaluation of the primary care for chronic diseases in the high coverage context of the Family Health Strategy

**DOI:** 10.1186/s12913-019-4737-2

**Published:** 2019-11-29

**Authors:** Kelly Cristina Gomes Alves, Rafael Alves Guimarães, Marta Rovery de Souza, Otaliba Libânio de Morais Neto

**Affiliations:** 1 Institute of Tropical Pathology and Public Health, Federal University of Goiás, Goiânia, Goiás Brazil; 2 Department of Medicine, Federal University of Tocantins, Palmas, Brazil

**Keywords:** Primary health care, Family practice, Primary care model, Noncommunicable disease, Chronic diseases, Chronic care model, Evaluation of care, Quality of care, Brazil

## Abstract

**Background:**

This cross-sectional study evaluated the adequacy of the Family Health Strategy for the primary care model for chronic noncommunicable diseases and the changes that occurred between the two cycles of external evaluations of the National Program for Improving Access and Quality of Primary Care, which took place in 2012 and 2014, in the higher coverage context of the Family Health Strategy of Brazil, in the state of Tocantins, Brazil.

**Methods:**

The data source contained information on the infrastructure of the 233 Primary Health Units and on the work process of 266 health teams. The Principal Component Analysis for Categorical Data technique and the McNemar chi-squared statistical test for comparing paired samples were used, and a significance level of 5% with a 95% Confidence Interval was used.

**Results:**

The analysis identified a low proportion of dispensing of medications for the treatment of chronic disease in both cycles. There was a significant increase in seasonal influenza vaccination, in the number of sterilization, procedure, dressings and inhalation rooms. There was a small but significant reduction in the materials for cervical cancer screening, although they are available in almost 90.0% of the PHUs. More than 70.0% of the health teams carried out additional health education activities, encouraged physical activity, registered schoolchildren with health needs for monitoring, evaluated user satisfaction and user referral.

**Conclusions:**

The findings of this study highlighted the improvement of the structure of the Primary Health Units, but identified a low provision of medicines to treat chronic diseases. The health promotion was performed as the main work process tool of family health teams, but it was little focused on intersectoral actions and on actions with the population in the area or on the empowerment of users through self-management support for chronic diseases. Furthermore, it is critical that the Family Health Strategy in Tocantins be organized and focused on the care of chronic diseases to improve and adapt itself to a primary chronic care model.

## Background

Noncommunicable diseases (NCDs) were responsible for 41 million (71.0%) deaths worldwide in 2016, with four main groups of causes: cardiovascular diseases (44.0%), neoplasms (22.0%), chronic respiratory diseases (9.0%) and diabetes (4.0%); approximately 44.0% of these deaths occurring in people between 30 and 70 years of age. More than 80.0% of these premature deaths occurred in low- and middle-income countries where the disease burden is greater, resulting in large economic losses for the countries. Premature deaths can be avoided by reducing the major modifiable risk factors, such as smoking, harmful alcohol use, unhealthy eating and physical inactivity [1]. In Brazil, proportional mortality due to NCDs was responsible for 59.6% of all deaths in 1990, 72.0% in 2011 and 75.8% in 2015 [2, 3]. Almost half of the Brazilian population (45.1%) reported having at least one chronic disease, with hypertension being the most frequently mentioned disease (21.4%) in the National Health Survey (*Pesquisa Nacional de Saúde*), conducted in 2013 throughout Brazil [4].

The World Health Organization [1] proposed a set of systematized interventions in the Global Plan to combat NCDs and reduce these deaths, with the goal of reducing them by one-third by 2030. The Plan proposes the strengthening of a health system directed toward integral care for people with NCDs. This plan especially includes the restructuring of Primary Health Care, the purpose of which is prevention, early detection, appropriate treatment, reduction of the main factors, risk management and the continued management of cases and people at high risk for major diseases; the goal of these aims is to prevent complications such as hospitalizations and premature deaths, as well as reduce financial expenses [5].

In Brazil, since the creation of the Brazilian Nation Health System (*Sistema Único de Saúde* - SUS) in 1988, the structuring and expansion of Primary Health Care has been carried out through the Family Health Strategy (FHS). The FHS performs activities through family health teams in Primary Health Units (PHUs) aimed at serving the target population living near the PHUs, known as ascribed population [6]. Substantial investments at the federal, state and municipal levels have led to a progressive increase in family health teams, with a population coverage in the country of 53.4% ​​in 2013. The state of Tocantins, in the northern region of the country, has the highest coverage (93.6%) among the Brazilian states [7].

In 2011, the Strategic Action Plan to Tackle Chronic Noncommunicable Diseases in Brazil 2011–2022 was instituted, with three important intervention axes: surveillance, information, evaluation and monitoring; health promotion; and integral care [8]. In the same year, to promote the expansion of coverage and increase the scope of the Primary Health Care actions [9], the National Primary Care Policy (*Política Nacional de Atenção Básica*) was updated, emphasizing the prevention and organization of primary care for NCDs patients [6]. In this context, the National Program for Improving Access and Quality of Primary Care (*Programa Nacional de Melhoria do Acesso e da Qualidade da Atenção Básica* - PMAQ-AB) was created with the aim of evaluating and promoting the improvement of Primary Health Care through changing the healthcare model, with an emphasis on prevention and integral care for NCDs [10]. The PMAQ-AB has national coverage, is linked to financial incentives for municipal management with the adhesion of family health teams to the Program and has three phases: adherence and contracting (phase 1), certification (phase 2) and recontracting (phase 3). In phase 2, the external evaluation is responsible for collecting data on the PHUs infrastructure, disease prevention, health promotion and clinical management of various diseases, including NCDs, as well as user satisfaction [11].

The PMAQ-AB is a data source with great potential to assess the capacity of the health teams to tackle NCDs in the Primary Health Care context. Furthermore, it may be a data source that can be used to assess the adequacy of the strategies used in the FHS according to the theoretical model of chronic care, such as the chronic care model proposed by Wagner [12]. Although the PMAQ-AB has not been structured to exclusively evaluate health actions directed toward NCDs, it is important to verify whether the variables contained in it allow the essential elements of a primary care model for NCDs to be evaluated, as well as the identification of those that better explain this model. It is also important to determine whether there were improvements in primary care for NCDs after the interventions of the PMAQ-AB and which variables are more capable of identifying the changes between the cycles that were evaluated. The aim of this study was to evaluate the adequacy of the structure of PHUs and the work process of family health teams in the FHS according to the primary care model for NCDs and also to examine the changes that occurred between the two cycles of external evaluations of the PMAQ-AB, which took place in 2012 and 2014, in the FHS of the state of Tocantins, Brazil.

### Methods

#### Study design and population

This was a cross-sectional analytical study in which the data source was the national database of the PMAQ-AB, which contains information on the infrastructure of the PHUs and the work process of the family health teams. This database was generated by data collection from the external evaluation, phase 2 of the Program, based on two cross-sectional studies conducted in Cycle 1 and Cycle 2, in 2012 and 2014, respectively. Data collection in both cycles was managed by the general coordinator of the program located in the Primary Care Department (*Departamento de Atenção Básica* - DAB) of the Ministry of Health in partnership with several teaching and research institutions in Brazil. The coordination of the data collection in the state of Tocantins was performed by Fiocruz-Rio de Janeiro, and the local coordination of the two cycles was delegated to the first author of this article. The national studies were approved under authorization No. 32012/2012 and No 357.974/2013 of the Research Ethics Committee of the National School of Public Health.

The state of Tocantins is the newest of the 26 states in Brazil, has 139 municipalities and is located in the Northern Region. It is part of the Amazon region, has extensive rural areas with a low population density and was one of the states with the highest adherence to the PMAQ-AB in Cycle 1 and Cycle 2. There were 400 family health teams in July 2012 and 475 teams in July 2014 [13]. The study population was composed of the PHUs and family health teams that adhered to the PMAQ-AB in both Cycle 1 and Cycle 2. In Cycle 1, 291 PHUs were studied, and 306 teams were interviewed, with 261 PHUs and 361 teams in Cycle 2. After the exclusion of the PHUs and teams that did not participate in both cycles, 233 PHUs and 266 teams working in 90 (64.75%) municipalities of the state that participated in the two cycles were included in the data analysis of this study.

#### Logical model, data collection and selection of the variables

To date, the PMAQ-AB has submitted three external evaluation cycles for the years 2012, 2014 and 2017, and the data for Cycle 1 and Cycle 2 are already available in the DAB database. Its data collection instruments are composed of three modules, which are broken down as follows: module I - observation in the PHUs regarding the infrastructure, equipment, materials and inputs; module II - interview with professionals at the PHUs regarding the performance of the family health teams in the dimensions of Primary Health Care management, health promotion, prevention and integral care for users; and module III - interview with users at the PHUs regarding their satisfaction with the care received. Other modules were incorporated after Cycle 1. In this study, the first two modules were considered.

**Fig. 1:**
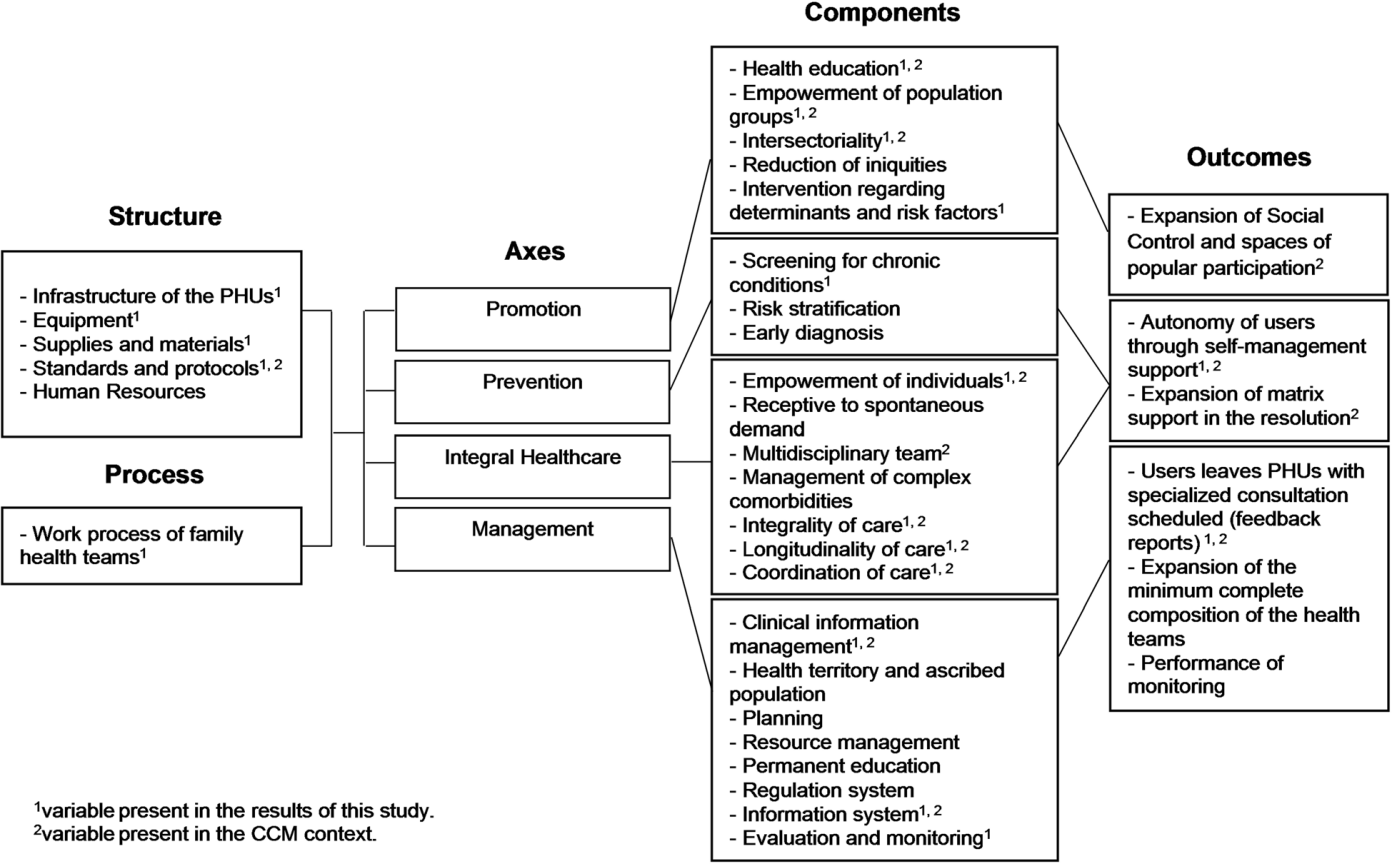
Logical model of structure and work process for NCDs primary care from the PMAQ-AB evaluation

Although PMAQ-AB has not been structured to exclusively evaluate health actions focused on NCDs, it is a data source with great potential for assessing primary care for NCDs in the FHS and it is the largest national database for assessing FHS. To evaluate the adequacy of primary care for the NCDs care performed by the FHS teams, from the data contained in modules I and II of the PMAQ-AB, a logic evaluation model was constructed. This logical model used the health service evaluation models proposed by Donabedian [14], the chronic care model of Wagner [12] and the model of Hartz and da Silva [15] as the theoretical framework. The logical model developed for the study (Fig. 1) consists of the following components: (i) structure of the PHUs, (ii) work process of the family health teams, and (iii) outcomes based in the organization of the family heath teams [14]. This study prioritized the evaluation of the adequacy of the primary care provided by the FHS teams directed toward NCDs using the framework of Wagner [12] systematized in the chronic care model. The elements of the chronic care model include the design of service provision, decision support, the clinical information system, self-management support and community resources [16]. Some variables obtained from the external evaluation of the PMAQ-AB are contained in the elements of the chronic care model. Especially those related with actions for self-management support groups for NCDs, physical activity, activities in schools, registration of schoolchildren with health needs, health education, evaluation of user satisfaction, protocols, local health council and spaces of public participation, monitoring and analysis of indicators and health information, matrix support in the resolution of complex cases, specialized consultation scheduled, records of users and family.

After construction of the logical model, the model adequacy variables corresponding to the structure and process were selected in the PMAQ-AB database for state of Tocantins. Thus, the variables related to the primary care for NCDs in the FHS, according to the logical model, were collected. It was not possible to collect outcome variables directly related to users, such as blood glucose level or hypertension control. The outcome variables selected were those that demonstrated the level of organization of the work process of family health teams for tackling NCDs. The variables used in the study were constructed from two databases: a database with information on the infrastructure of the 233 PHUs responsible for the same ascribed population in the two cycles (2012 and 2014), and another with information on the work process of the 266 teams that operated in these 233 PHUs during the period.

The same variables of Cycle 1 and Cycle 2 were selected, and those common to the two cycles were used. In the variables with alterations in the categories of the responses, compatibilization was performed to be able to compare the cycles, excluding the variables that differed between them and excluding those that were not directly related to NCDs care and those with 10.0% or more missing data. A total of 55 variables of the PHUs structure were used for Cycle 1 of the PMAQ-AB, along with the corresponding ones present in Cycle 2, as well as 49 variables of the work process of the family health teams for the two cycles, resulting in a total of 104 variables in Cycle 1 and 104 variables in Cycle 2. The selected variables were, therefore, those that included the dimensions present in the logical model.

#### Statistical analysis

Initially, descriptive analysis by absolute (*n*) and relative (%) frequency was performed for each of the variables of the two cycles for the evaluation of consistency. The Principal Component Analysis for Categorical Data (CATPCA) technique was used to identify the variables or group of variables that best explained the variance of the original variables and reduce the variables for analysis of the variation between the two cycles of PMAQ-AB [17, 18]. The CATPCA transforms qualitative variables into quantitative variables by means of a technique called optimal scaling, so that the transformation maximizes the responsible variance in the data set, thus creating a two-dimensional scale [19]. This technique is similar to nonlinear principal component analysis (NLPCA). The aim of this analysis was to reduce the number of variables without compromising the information necessary for the study [17, 18, 20].

For CATPCA, a tetrachoric correlation matrix was first performed among all the variables of the model, which was used to conduct a factorial analysis of binary variables [21, 22]. This type of correlation is indicated as a substitution for Pearson’s correlation to investigate the bivariate relationship between two dichotomous variables [21–25]. Variables with correlation coefficients lower than 0.3 (r_t_ <  0.3) with any other variable of the matrix were excluded from the analysis. Next, multiple imputation of the missing values was carried out for each variable using the modal value of the respective variable [26]. To determine the number of principal components (PC) to be extracted in the analysis, the eigenvalue criterion and Cronbach’s alpha were used, and the extracted components were those that presented eigenvalues greater than 1 [17, 18, 27] and presented Cronbach’s alpha values greater than 0.6 as acceptable reliability [26]. Variables with a factor loading ≥0.4 were considered to belong to each component [17, 18]. Two CATPCA models were performed for the structure of PHUs (Cycles 1 and Cycle 2) and two models for the variables of the work process (Cycles 1 and Cycle 2). After extraction of the main components, box plot plots and descriptive analysis of the component scores were performed. Comparison of scores between the two cycles (Cycle 1 versus Cycle 2) was not possible due to differences in the retained variables and factor loadings in the CATPCA models of the two cycles.

To analyze the adequacy of the FHS according to the primary care model for NCDs, the variables of the components from the CATPCA were used, and the framework of inference of adequacy proposed by Habicht et al. [28] was employed. Habicht’s theoretical model of program/intervention evaluation is based on two axes of evaluation classification: intervention performance and impact. Both analyses are based on three type of inferences: adequacy, plausibility, and probability. For adequacy assessment, provision, coverage and impact indicators can be used. In the case of this study, the indicators of health service provision by the FHS were used. Comparison between the differences in the items of the variables retained in the CATPCA models (adequacy of the structure of the PHUs and adequacy of the work process) between Cycle 1 and Cycle 2 of the PMAQ-AB was carried out using the McNemar chi-squared statistical test for comparison of paired samples, considering a significance level of 5 and 95% Confidence Interval [29]. The data were analyzed using the Statistical Package for the Social Sciences, version 24.0.

## Results

### Descriptive analysis

Additional file 1 shows the descriptive analysis of the 104 variables of Cycle 1 and the corresponding variables of Cycle 2 of the PMAQ-AB, which were used in this study to evaluate the adequacy of the structure and work process carried out in the FHS according to the primary care for NCDs and the changes that occurred between these two cycles in the state of Tocantins.

### PHUs analysis

The CATPCA model for the structural variables of the 233 PHUs of Cycle 1 included 54 of the 55 variables initially investigated. One variable (nursing consultations) was excluded because it did not present any variation in values, impeding the tetrachoric correlation analysis. The other variables presented a correlation coefficient (r_t_) ≥ 0.3 with any other variable analyzed. Based on the eigenvalues, three PC were extracted from this analysis: PC1, PC2 and PC3. For Cycle 2, the CATPCA analysis included 54 of the 55 variables. One variable (table for clinical examination) was excluded because it did not present any variation. The other variables presented a r_t_ ≥ 0.3 with any other variable analyzed. Based on the eigenvalues, three components also were extracted from this analysis: PC1, PC2 and PC3 (Additional file 2).

For Cycle 1, the PC1 could be interpreted as medications for the treatment of NCDs; the PC2 could be interpreted as materials for cervical cancer screening, and the PC3 could be interpreted as the provision of vaccinations and infrastructure. For Cycle 2, the variables that remained in the model were similar to those in Cycle 1 and received the same denominations as the Cycle 1 due to the similarity of the variables retained. The CATPCA summarized a set of 55 structural variables on a scale of 32 two-dimensional variables (24 Cycle 1 and Cycle 2; 3 only Cycle 1, and 5 only Cycle 2) that could be used in the minimum evaluating of the structural adequacy of the PHUs for primary care for NCDs. Table 1 summarizes the variables explaining the PHUs structure belonging to Cycle 1 and Cycle 2 of the PMAQ-AB.

**Table 1:** Factor loading of the PHUs structure. CATPCA model (*n* = 233)

Principal Components	Cycle 1	Cycle 2
	Factor loading	
PC1		
Medications for the treatment of NCDs	0.797	0.638
Prednisone	0.797	0.638
Salbutamol	0.679	0.807
Ipratropium bromide	0.678	0.804
Captopril	0.439	0.900
Beta blockers	0.816	0.936
Hydrochlorothiazide	0.829	0.873
Losartan	0.646	0.778
Simvastatin	0.545	0.695
Glibenclamide	0.881	0.876
Metformin	0.830	0.895
NPH Insulin	0.771	0.864
Regular Insulin	0.735	0.814
Fenoterol	0.714	^a^
Beclomethasone	^a^	0.469
PC2		
Materials for cervical cancer screening		
Speculum	0.711	0.878
Endocervical brush	0.770	0.844
Ayre spatula	0.816	0.813
Slide clamp	0.715	0.718
Glass blade with matte side	0.653	0.692
Blade holder	0.463	0.765
Gynecological table	0.515	^a^
Light focuser	0.435	^a^
Provide nursing consultations	^a^	0.401
PC3		
Provision of vaccinations and infrastructure		
Seasonal influenza vaccine	0.400	0.519
Vaccination	0.416	0.522
Vaccination room	0.477	0.534
Sterilization room	0.559	0.429
Team with Internet access	0.433	0.418
Vehicle for external activities	0.525	0.422
Procedures room	^a^	0.509
Dressings room	^a^	0.477
Inhalation room	^a^	0.430

*Abbreviations*: *PHUs* primary care units, *NPH* Neutral Protamine Hagedorn, *NCDs* chronic noncommunicable diseases, *PC* Principal Components

Definitions of symbols: ^a^factor loading < 0.4, therefore, not extracted by the CATPCA model to integrate the components in this Cycle

The variables extracted from the CATPCA explained the variability of the primary care for NCDs with respect to the structure of the PHUs, with the exception of standards and human resources that had no variables in the final CATPCA model, and the explanatory variables are the axes of the logical model (Fig. 1). Analysis of the adequacy of the structure of the PHUs (Table 2) identified a low proportion of dispensing of sufficient quantity of medications for the treatment of NCDs in Cycle 1 and Cycle 2. These medications were available in less than half of all PHUs evaluated. Only captopril showed a significant increase during the study period. There was a small but significant reduction in the materials for cervical cancer screening, although they are available in almost 90.0% of the PHUs. Seasonal influenza vaccination increased significantly and was available in 49.8% of the PHUs in Cycle 1 and 84.5% in Cycle 2 as well as the percentage of PHUs with sterilization room.

**Table 2:** Variation of structure variables of PHUs retained by the CATPCA according to the logical model (*n* = 233)

Elements of logical model	Variables	Cycle 1		Cycle 2		Variation	*p*-value^†^
		n	%	n	%	% (95% CI)	
	PC1						
	Medications for the treatment of NCDs						
Supplies and materials	Prednisone	102	43.8	58	24.9	−18.9 (−27.1; − 10.3)	< 0.001
	Salbutamol	81	34.8	78	33.5	−1.3 (− 9.8; 7.3)	0.798
	Ipratropium bromide	83	35.6	76	32.6	−3.0 (− 11.5; 5.6)	0.457
	Captopril	39	16.7	102	43.8	27.1 (18.9; 34.7)	< 0.001
	Beta blockers	125	53.6	110	47.2	−6.4 (−15.4; 2.6)	0.096
	Hydrochlorothiazide	122	52.4	93	39.9	−12.5 (−21.2; −3.4)	0.001
	Losartan	69	29.6	76	32.6	3.0 (−5.4; 11.4)	0.500
	Simvastatin	55	23.6	56	24.0	0.4 (− 7.3; 8.2)	1.000
	Glibenclamide	126	54.1	100	42.9	−11.2 (−20.0; −2.1)	0.002
	Metformin	124	53.2	101	43.3	−9.9 (−18.7; −0.8)	0.010
	NPH Insulin	90	38.6	92	39.5	0.9 (−7.9; 9.6)	0.896
	Regular Insulin	82	35.2	83	35.6	0.4 (−9.3; 8.2)	1.000
	Fenoterol^a^	87	37.3	20	8.6	−28.8 (−35.8; −21.4)	0.008
	Beclomethasone^b^	22	9.4	31	13.3	3.9 (−2.0; 9.7)	0.149
	PC2						
	Materials for cervical cancer screening						
	Speculum	221	94.8	212	91.0	−3.8 (−8.8; 0.9)	0.093
	Provide nursing consultations^b^	233	100.0	231	99.1	−0.9 (−3.1; 0.9)	0.500
	Endocervical brush	221	94.8	207	88.8	−6.0 (−11.2; −1.0)	0.013
	Ayre spatula	221	94.8	208	89.3	−5.5 (−10.7; −0.6)	0.019
	Slide clamp	222	95.3	209	89.7	−5.6 (−10.6; −0.8)	0.019
	Glass blade with matte side	220	94.4	211	90.6	−3.8 (−9.2; 1.0)	0.108
	Blade holder	201	86.3	204	87.6	1.3 (−4.9; 7.8)	0.766
Equipment	Gynecological table^a^	222	95.3	225	96.6	1.3 (−2.1; 4.7)	0.969
	Light focuser^a^	220	94.4	224	96.1	1.7 (−2.3; 5.9)	0.861
	PC3						
	Provision of vaccinations and infrastructure						
Supplies and materials	Seasonal influenza vaccine	116	49.8	197	84.5	34.7 (26.5; 42.3)	< 0.001
	Provide vaccination	220	94.4	216	92.7	−1.7 (−6.4; 2.9)	0.454
Infrastructure	Sterilization room	114	48.9	166	71.2	22.3 (13.5; 30.7)	< 0.001
	Vaccination room	212	91.0	208	89.3	−1.7 (−7.3; 3.8)	0.481
	Procedures room^b^	111	47.6	204	87.6	40.0 (31.9; 47.2)	< 0.001
	Dressings room^b^	168	72.1	210	90.1	18.3 (11.0; 24.9)	0.008
	Inhalation room^b^	33	14.2	177	76.0	61.8 (54.1; 68.2)	< 0.001
Equipment	Team with Internet access	104	44.6	108	46.4	1.8 (−10.7; 7.3)	0.659
	Vehicle for external activities	140	60.1	128	54.9	−5.2 (−14.0; 3.8)	0.134

*Abbreviations*: *PHUs* primary care units, *CI* Confidence Interval, *NPH* Neutral Protamine Hagedorn, *PC* principal component

Definitions of symbols: ^a^ = Only Cycle 1; ^b^Only Cyle 2; ^†^ = McNemar test

It is worth highlighting those variables that varied in the period, even though they were not retained in the same PCs for the same cycles. The variables procedures room, dressings room and inhalation room which also increased significantly in the period studied.

Additional file 2 and Additional file 4: Figure S1 show the descriptive analysis of scores extracted from CATPCA of the PHUs structure. The median scores of the components were: PC1 (0.050 – Cycle 1 and − 0.485 – Cycle 2), PC2 (− 0.230 – Cycle 1 and 0.290 – Cycle 2) and PC3 (− 0.120 – Cycle 1 and 0.080 – Cycle 2).

### Work process analysis

The CATPCA performed for the work process variables of the 266 health family teams of Cycle 1 and Cycle 2 included 49 investigative variables, all of which presented a correlation coefficient (r_t_) ≥ 0.3 with any other variable analyzed. According to the eigenvalue criterion > 1 and Cronbach’ alpha ≥ 0.6, two components were extracted from the analysis of Cycle 1: PC1 and PC2. For Cycle 2, the results of the CATPCA model presented many differences in relation to Cycle 1 and also allowed the extraction of two components: PC1 and PC2 (Additional file 3).

For Cycle 1, the PC1 could be interpreted as health promotion and the PC2 could be health education for people with diabetes and hypertension. For Cycle 2, the PC1 could be interpreted as health promotion and health site analysis and PC1 as health education and user referral. The CATPCA summarized a set of 49 variables related to the work process of the family health teams on a scale of 18 two-dimensional variables (only 6 variables common to Cycle 1 and Cycle 2; 5 only Cycle 1 and 7 only Cycle 2) - that can be used in the minimum evaluation of the work process of the teams regarding primary care for NCDs. Table 3 summarizes the explanatory variables of the work process of the family health teams belonging to Cycles 1 and Cycle 2 of the PMAQ-AB.

**Table 3:** Factor loading of the work process of the family health teams. CATPCA model (*n* = 266)

Principal Components	Factor loading
Cycle 1	
PC1	
Health promotion	
Health education for women	0.532
Health education for older adults	0.595
Health education addressing healthy eating^a^	0.561
Health education for men	0.472
Document that proves the performance of health education	0.626
Registration of schoolchildren with health needs^a^	0.412
Evaluation of user satisfaction^a^	0.410
Provide actions for groups of people with hypertension^a^	0.484
Provide actions for groups of people with diabetes^a^	0.453
Provide actions for groups of self-management support for NCDs^a^	0.502
Registration of bedridden people^a^	0.416
PC2	
Health education for people with diabetes and hypertension	
Document that proves the performance of health education	0.431
Provide actions for groups of people with hypertension	0.521
Provide actions for groups of people with diabetes	0.459
Cycle 2	
PC1	
Health promotion and health site analysis	
Health education schedule	0.400
Physical activities	0.414
Registration of schoolchildren with health needs^a^	0.410
Evaluation of user satisfaction^a^	0.465
Provide actions for women’s groups (cancer prevention)	0.464
Provide actions for groups of people with obesity	0.433
Provide actions for groups of people with hypertension^a^	0.548
Provide actions for groups of people with diabetes^a^	0.530
Protocol for priority home visits	0.431
Registration of bedridden people^a^	0.531
Management provides information for health situation analysis	0.474
PC2	
Health education and user referral	
Health education addressing healthy eating^a^	−0.407
Provide actions for women’s groups (cancer prevention)	0.508
Provide actions for groups of people with hypertension^a^	0.596
Provide actions for groups of people with diabetes^a^	0.596
User receives referral form to seek scheduling	0.409

*Abbreviations*: *NCDs* chronic noncommunicable diseases, *PC* Principal Components

Definitions of symbols: ^a^ = Variables also retained in some component of Cycle 1

Analysis of the adequacy of the work process of the family health teams (Table 4) showed that of the 18 variables extracted from the CATPCA in at least one Cycle, 10 did not present a significant difference between Cycle 1 and Cycle 2 of the PMAQ-AB, which highlighted the significant increase in health promotion actions. The family health teams carried out more activities of health education for the ascribed population.

**Table 4:** Variation of variables of work process retained by the CATPCA according to the logical model (*n* = 266)

Elements of the logical model	Variables	Cycle 1		Cycle 2		Variation	*p*-value^†^
		n	%	n	%	% (95% CI)	
Health education	Health education for women^a^	248	93.2	222	83.5	−9.7 (−15.3; −4.4)	0.008
	Health education for older adults^a^	245	92.1	223	83.8	−8.3 (−13.9; −2.8)	0.004
	Health education addressing healthy eating	232	87.2	203	76.1	−10.0 (− 17.4; − 4.4)	0.040
	Health education for men^a^	118	44.4	179	67.3	22.9 (14.6; 30.9)	< 0.001
	Document that proves the performance of health education^a^	218	82.0	198	74.4	−7.5 (−14.5; 0.5)	0.426
	Health education schedule^b^	205	77.1	239	89.8	12.8 (6.5; 19.0)	0.002
Intersectoriality	Registration of schoolchildren with health needs	62	23.3	92	34.6	11.3 (3.6; 18.8)	0.001
Empowerment of individuals	Evaluation of user satisfaction	75	28.2	143	53.8	25.6 (17.3; 33.3)	0.001
	Provide actions for groups of self-management support for NCDs^a^	169	63.5	169	63.5	0.0 (−8.1; 8.1)	0.849
Coordination of care	Registration of bedridden people	117	44.0	132	49.6	5.6 (−14.0; 2.8)	0.159
Intervention regarding determinants and risk factors	Provide actions for groups of people with diabetes	251	94.4	244	91.7	−2.6 (−7.1; 1.8)	0.781
	Physical activities^b^	136	51.1	187	70.3	19.2 (18.6; 19.8)	< 0.001
	Provide actions for women’s groups (cancer prevention)^b^	237	89.1	230	86.5	−2.6 (−3.0; 8.3)	0.425
	Provide actions for groups of people with obesity^b^	116	43.6	123	46.2	−2.6 (−11.0; 5.9)	1.000
	Protocol for priority home visits^b^	100	37.6	98	36.8	−0.8 (−8.9; 7.4)	0.921
Clinical information management	Management provides information for health situation analysis^b^	240	90.2	249	93.6	3.8 (−1.3; 8.2)	0.488
Integrality and Longitudinality of care	User receives referral form to seek scheduling^b^	113	42.5	142	53.4	10.9 (2.4; 19.2)	0.016

*Abbreviations*: *CI* Confidence Interval, *NCDs* chronic noncommunicable diseases

Definitions of symbols: ^a^ = Only Cycle 1; ^b^Only Cyle 2; ^†^ = McNemar test

It is worth highlighting the variables that presented significant difference despite not being retained in the same PC. The variables health education for men, registration of schoolchildren with health needs for monitoring, evaluation of user satisfaction, health education schedule, incentive to physical activity and user receives referral form to seek scheduling were the actions that increased significantly during the study period. The variables health education for women, for the older adults and for addressing healthy eating were the actions that decreased significantly during that period.

Table 5 summarizes the variables that changed, those that increased and decreased the adequacy percentages, and those which did not change between 1 and Cycle 2 of PMAQ-AB.

**Table 5:** Description of the variables that increased, decreased, and did not change between PMAQ-AB Cycles

Dimensions of the FHS	Variables		
	Increased	Decreased	Did not change
Structure of PHUs	Captopril	Prednisone	Salbutamol
	Seasonal influenza vaccine	Hydrochlorothiazide	Ipratropium bromide
	Sterilization room	Glibenclamide	Beta blockers
	Procedures room	Metformin	Losartan
	Dressings room	Fenoterol	Simvastatin
	Inhalation room	Endocervical brush	NPH Insulin
		Ayre spatula	Regular Insulin
		Slide clamp	Beclomethasone
			Speculum
			Provide nursing consultations
			Glass blade with matte side
			Blade holder
			Gynecological table
			Light focuser
			Provide vaccination
			Vaccination room
			Team with Internet access
			Vehicle for external activities
Work process of family health teams	Health education for men	Health education for women	Document that proves the performance of health education
	Health education schedule	Health education for older adults	Provide actions for groups of self-management support for NCDs
	Registration of schoolchildren with health needs	Health education addressing healthy eating	Registration of bedridden people
	Evaluation of user satisfaction		Provide actions for groups of people with diabetes
	Physical activities		Provide actions for women’s groups (cancer prevention)
	User receives referral form to seek scheduling		Provide actions for groups of people with obesity
			Protocol for priority home visits
			Management provides information for health situation analysis

*Abbreviations*: *PHUs* primary care units, *NPH* Neutral Protamine Hagedorn, *NCDs* chronic noncommunicable diseases

Additional file 3 and Additional file 5: Figure S2 show the descriptive analysis of scores extracted from CATPCA of the work process of family health teams. The median scores of the components were: PC1 (− 0.165 – Cycle 1 and 0.171– Cycle 2) and PC2 (− 0.066 – Cycle 1 and 0.063 – Cycle 2).

### Discussion

The CATPCA results identified patterns (principal components - PC) for evaluating the adequacy of structure of PHUs and the work process of the family health teams in the primary care for NCDs in the state of Tocantins, Brazil. Three patterns were identified in the PHUs analysis and two patterns in the work process analysis of family health teams. These PC were composed of the variables that showed the greatest variability among the PHUs and among the teams evaluated, and those that better explained the variability in each PMAQ-AB cycle. However, it is important to highlight the need for further studies of this same methodology, which could vary for each Brazilian state.

The variables of the PC varied much more among the PHUs than among the teams, as observed from the high factor loadings found for the PHUs. In the analysis of PHUs, the three components obtained by the CATPCA (medications for the treat of NCDs, materials for cervical cancer screening, and provision of vaccinations and infrastructure) were similar for the two cycles, which favored the comparison among all of them in the assessment of adequacy. For the teams, two components were obtained by the CATPCA (health promotion and health education for people with diabetes and hypertension for Cycle 1 and health promotion and health site analysis and health education and user referral for Cycle 2), with different variables, if compared in one cycle and another. Variables related to prevention, integral health care and NCDs management, according to the logical model, did not remain in the final CATPCA model because they did not have a sufficient factorial load; therefore, they were not compared between Cycle 1 and Cycle 2.

Evaluation of the structure of PHUs indicated the low availability of medications for the treatment of NCDs. The only structure variables that increased significantly in the studied period were the percentages of captopril and seasonal influenza vaccinations and the number of sterilization, procedures, dressings and inhalation rooms. The work process of family health teams showed significant improvement regarding health promotion activities.

Tocantins has the highest national coverage of the FHS [7], which is typical of underdeveloped regions such as the northern and northeastern regions of the country, where the Human Development Index (HDI), which is a proxy for socioeconomic conditions, is lower [30]. Increasing FHS coverage in these poorer regions, with the aim of reducing health inequities and increasing access to Primary Health Care [7], has not been sufficient to overcome the precarious conditions of the PHUs in these regions. They have the lowest scores of any region for infrastructure and lack consumables, medications and Information Technology (IT) equipment, such as computers and Internet access, with even worse findings for rural areas, indicating important social and structural inequalities in the municipalities [31], which it is not the case for the municipalities with lower FHS coverage that have a higher prevalence of adequate PHUs [32].

The availability of medications for the treatment of NCDs in the PHUs in Tocantins is below 50.0% for all types of medication studied and falls far short of the 80.0% recommended by the World Health Organization [33], with insulin present in less than 40.0% of the PHUs. Studies evaluating the PHUs in the entire country have also demonstrated an availability of medications for the treatment of hypertension and diabetes below 50.0% in Cycle 1 of the PMAQ-AB [34] and approximately 40.9% for the treatment of diabetes in Cycle 2 [32]. The free provision of medications for the treatment of NCDs has increased in the country since 2011 through the Popular Pharmacy Program and expanded the access of users [3]. However, several factors are related to adherence to treatment for hypertension and diabetes in Primary Health Care [35], including the proximity of the primary care source to the users of the health service, a conditional factor for people with diabetes who use insulin [36]. Furthermore, because Primary Health Care is well positioned to tackle NCDs as a gateway to the health system [37], the PHUs are care points that are strategically located close to the ascribed population and may promote greater access to medications for users with NCDs who attend the same primary care source over time.

Similarly, screening for cervical cancer can be carried out adequately and efficiently in PHUs [37]. The significant reduction of the screening materials identified in this study, despite having high availability in the PHUs of Tocantins, is worrying, since the maintenance of their adequacy over time is expected, especially considering that these materials are of low cost and low technological complexity. Similar studies have identified improvements in the quality of the screening due to the adequacy of the PHUs structure and the work process of the family health teams [38]. The adequacy of the PHUs structure across the country was of 50.0%, with missed opportunities for screening occurring in the presence of persistent problems in the structure and work process [39]. It is likely that the high prevalence in the provision of these materials was induced by PMAQ-AB [11] because the Program guarantees greater financial resources to the PHUs that present improvements in the structure and work process of family health teams. In addition, cervical cancer screening is a priority for FHS in the primary care context in Brazil [38, 39].

About PC3 (provision of vaccinations and infrastructure), the high rate of availability of the influenza vaccine in Tocantins is an important marker for the adequacy of the structure of the PHUs, since vaccinations against influenza and pneumonia have been shown to be effective in preventing complications and hospitalizations among users with chronic obstructive pulmonary disease [40]. The observation that the PHUs have a sufficient amount of available vaccines can be an important factor for high vaccination coverage in Tocantins. The state has one of the highest vaccination coverage rates in the country (89.1%) according to findings from a similar study, which showed a national rate of 61.8% [41] and influenza vaccination coverage in priority groups exceeding 70% between 2010 and 2012 [42]. International studies have reported lower influenza vaccination rates in a Primary Health Care service of the United States of America [43] and ones similar to those of Tocantins among users participating in a study performed in the city of Birmingham in the United Kingdom [44].

An improvement in the infrastructure of the PHUs in Tocantins can be considered, which increased the percentage of sterilization, procedures, dressings and inhalation rooms in the PHUs in Cycle 2 of the PMAQ-AB. Some national studies, using data from the PMAQ-AB, related the improvement of the infrastructure of the PHUs to the increased proportion of some types of rooms to perform procedures and services for the users [31, 32, 34]. The improved infrastructure of the PHUs was probably driven by the increase in financing for construction and PHUs renovation through the PHUs Requalify Program [31], which began in 2010 throughout the country. In addition to the sterilization room, the findings of this study highlighted other important variables that did not present a significant difference in the period but were in the final CATPCA model, such as the vaccination room and provide vaccination, which are considered to be quality markers of the PHUs structure in the country [31]. This important result found in the statistical approach used in this study that can be useful to evaluate the adequacy of structure in other contexts.

Despite an absence of significant differences in the period under study, lack of Internet access for just under 50.0% of the family health teams in the PHUs investigated is worrisome, as there is evidence confirming positive results from the use of IT in primary care for NCDs. Overall, this finding reflects the incipient process of IT incorporation into Primary Health Care in Brazil in terms of structure, with 41.9% of the family health teams working in contexts that present low levels of IT [45]. The use of IT is fundamental in redesigning the primary care model for NCDs, especially considering its contribution to decision support and the implementation of clinical information systems [46]. The implementation is mainly carried out through records and adherence to clinical protocols [47] and it is fundamental for improving the performance of the health system. In a study carried out in 22 European countries, the need to improve the information infrastructure in the Primary Health Care of the countries was highlighted to achieve these objectives [48]. Strategies such as the use of reminders regarding medication treatment [35] and provision of the influenza vaccination [49] through interactive voice response (IVR) have proven to be positive. Furthermore, there is an association between IT incorporation and quality of care by family health teams, with better quality certification results in the PMAQ-AB [45].

Although the analysis of the work process of family health teams did not result in PCs with equal variables between cycles, as it happened in the PHUs analysis, it is worth highlighting those variables that showed significant differences in adequacy between Cycle 1 and Cycle 2 of PMAQ-AB. Analysis of the work process indicated health promotion as a key point in the work of these teams. The variables that significantly increased beetwen cycles were health education for men, registration of schoolchildren with health needs, evaluation of user satisfaction, health education schedule and physical activities and user receives referral form to seek scheduling.

These findings assume that family health teams are seeking to create greater links with the community through activities performed outside the PHUs environment, especially as schoolchildren were the target of the actions of one third of the teams in Cycle 2. This finding may be related to the advances of the School Health Program in 2008 [50], which encouraged health teams to visit schools and carry out health promotion activities among schoolchildren.

In a similar study using the PMAQ-AB, the data did not differ significantly from the findings of the present study. The majority of the family health teams reported carrying out health promotion actions, such as school activities and physical activity [51]. The findings of this study regarding health promotion are consistent with results from other studies in which health promotion was also identified as an important strategy in chronic care model evaluation contexts, with improvements in health education processes [52], physical activity [53] and satisfaction of the users [54]. Brazilian studies evaluating the chronic care model in the FHS identified improvements in links with the community [55] and positive changes in the care performed by the teams [56].

Regarding health education, the findings of this study do not allow an evaluation of the nature of the health education carried out by the teams. It is not known whether these actions involved potent educational processes to encourage the change of behavior of the users or merely informed them about the damage of certain habits that are harmful to health. It is known that the health education and health promotion programs offered in PHUs are directed more toward adults and elderly individuals, usually users with hypertension and diabetes [57], which justifies the high percentage of health teams developing activities for these groups in Tocantins in the two cycles of the PMAQ-AB. Furthermore, based on the National Health Survey (*Pesquisa Nacional de Saúde*), more than 80.0% of users with hypertension and diabetes who used public or private health services were given advice regarding adequate diet, body weight and the practice of physical activity. However, adherence to beneficial practices such as healthy eating and physical activity was not greater among these users, although they prioritized avoiding certain harmful habits, such as the use of tobacco products. This finding showed that these specific groups preferred to avoid harmful habits than to adhere to healthy practices [58].

The variables of health education for women, for older adults and for addressing healthy were those that decreased in the period. It is likely that family health teams have performed fewer health education actions for those ones in order to have more time to target these actions to men users. Historically, Primary Health Care in Brazil has been little directed to men’s health, with greater coverage for children, women and the elderly [59, 60]. The increase in health education for men may be an indicator of improved access for this population to the FHS.

The variable user receives referral form to seek scheduling is an important variable related to care coordination, which can become more effective when access to scheduling is guaranteed, even if scheduled by the user himself or by the PHUs. It is known that referral of users can be more effective if requested by family health teams and if the waiting time of the user to schedule appointments or specialized exams is shorter as possible [9].

Even though there was no significant difference between the PMAQ-AB cycles, self-management support is an important variable for evaluating the adequacy of the primary care for NCDs. However, this variable was not performed by almost 40.0% of the family health teams in the two PMAQ-AB cycles. Self-management support is one of the key components of the chronic care model that aims to improve chronic care in the Primary Health Care and to contribute to the redesign of health systems aimed at NCDs [60].

This study did not intend to investigate the possible interactions between structure and process to achieve expected outcomes in relation to the primary care model for NCDs. However, the adequacy of the structure is an element that contributes in favor of the work process of the family health teams in their various work contexts. According to the Donabedian quality evaluation model, a good structure increases the likelihood of a good process, which in turn increases the likelihood of a good result [14]. A study in South Africa found that, regardless of structure, a good process mediated the relationship between a good structure and a good outcome in the Primary Health Care model in South Africa [61]. In fact, PHUs with significant structural deficiencies make it difficult to retain professionals in the work process of the family health teams [62, 63]. Furthermore, the final analysis showed that the deficiencies can make it impossible to implement the necessary advances in the primary care model, with an emphasis on NCDs.

There are a number of limitations to this study. Of particular concern was that the database consisted of secondary data and there were too many variables to choose for the study. The variable human resource was excluded because it presented a high percentual of missing data. This study prioritized those variables according to the theoretical framework for primary care for NCDs, and systematized them in the logical model. In addition, it was not possible to collect outcome variables directly related to the user because they are not available in database. It was not possible to evaluate the adequacy of the FHS as a whole and make inferences about it due to lack of outcome variables directly related to the user. However, it was possible to evaluate in this study the adequacy of the structure and work process of family health teams to primary care for NCDs in the high coverage context of the FHS. Nevertheless, the PMAQ-AB database is currently the largest national FHS database and it has great potential for use in primary care evaluation research. Furthermore, PMAQ-AB is a program with voluntary adherence linked to financial incentive, which makes it possible that the teams that adhered were those with the best performance. There was a change in the external evaluation instrument, reducing the data analysis to the variables belonging to the two PMAQ-AB cycles. Moreover, the comparison between Cycles 1 and Cycle 2 in this study caused information losses related to those that did not participate in the two cycles. But this was minimized by the fact that Tocantins presented one of the highest rates of adherence to the Program in the country, encompassing almost all the PHUs and family health teams of the state.

Although some studies have used data from PMAQ-AB [31, 32, 34, 38, 39, 41, 45, 51, 53, 59], this is the first one that evaluated the structure of PHUs and the work process of family health teams for NCDs primary care using the CATPCA method. This approach was chosen because the PMAQ-AB data is categorical and a method was needed to reduce a large number of variables and identify the most powerful indicators explaining the variability of variables between PMAQ-AB cycles.

The PMAQ-AB may have led to improvements in the overall organization of the work process of the family health teams included in this study, especially regarding health promotion. However, more significant process changes may require additional time for evaluation, and further studies are needed incorporating data from the new PMAQ-AB cycles.

## Conclusions

The improvement of the PHUs infrastructure may have been important in the provision of rooms, influenza vaccine and materials for cervical cancer screening. However, the low supply of medicines undoubtedly constitutes an important barrier to be overcome in the FHS. The health promotion, especially health education actions and physical activity promotion, were the main work tools of family health teams. The family health teams need to expand their health promotion actions with greater emphasis on intersectoral actions beyond those carried out at school activities.

The structure of PHUs and the work process of family health teams have low adequacy, that is, they do not have enough power to tackle chronic diseases in the FHS. This low adequacy was due to the following problems: low availability of medicines for NCD, low use and access to IT, lack of substantial intersectoral actions of health promotion, fragile integrality and longitudinality of care regarding referral of users to experts and low actions for empowerment of the users through self-management support. All these variables have great potential for evaluating primary care for NCDs because they were widely used in studies that evaluated CCM.

The methodology and results of this study can be useful to improve the data collection instrument of PMAQ-AB. It can orient the revision of which variables should remain, be introduced or withdrawn in the PMAQ-AB questionaire and which ones better capture the care model for NCDs. Finally, the methodology used may support the Brazilian Ministry of Health in reviewing the PMAQ-AB data collection instrument, making it more powerful to evaluate the primary care model for NCDs implemented in the municipalities. And more importantly, can identify the limitations of the care model implemented by the FHS and support it in implementing actions that strengthen NCDs care in the territory where they operate.

## Supplementary information

**Additional file 1. **Description of the variables of the PHUs (*n* = 233) and the family health teams (*n* = 266).

**Additional file 2.** Factor loading of the variables of the PHUs structure.

**Additional file 3.** Factor loading of the variables of the work process of family health teams.

**Additional file 4: Figure S1.** Descriptive analysis of principal component scores between Cycles 1 and 2 for PHUs. Definitions of abbreviations: PHUs: primary care units; PC1: Medications for the treatment of NCDs; PC2: Materials for cervical cancer screening; PC3: Provision of vaccinations and infrastructure.

**Additional file 5: Figure S2.** Descriptive analysis of principal component scores between Cycles 1 and 2 for family health teams. Definitions of abbreviations: Cycle 1 - PC1: Health Promotion; PC2: Care for groups with diabetes and hypertension; Cycle 2 - PC1: Health promotion and health site analysis; PC2: Health education and user referral.

## Data Availability

The datasets analysed during the current study are available in the Departament of Primary Care (Departamento de Atenção Básica - DAB) repository, https://aps.saude.gov.br/ape/pmaq. To select data from sctructure of primary health units: 1. Select Ciclo 1 [Portuguese]; 2. Select Microdados da avaliação Externa [Portuguese]; 3. Then, choose Módulo I UBS [Portuguese]; 4. Finally, choose UBS Tocantins [Portuguese]. Do it the same for Ciclo 2 [Portuguese] to download the dataset. To select data from family health strategy teams: 1. Select Ciclo 1 [Portuguese]; 2. Select Microdados da avaliação Externa [Portuguese]; 3. Then, choose Módulo II Equipe [Portuguese]; 4. Finally, choose Equipe Tocantins [Portuguese]. Do it the same for Ciclo 2 [Portuguese] to download the dataset.

## Abbreviations

CATPCA: Principal Component Analysis for Categorical Data
FHS: Family Health Strategy
IT: Information Technology
NCDs: Chronic noncommunicable diseases
PC: Principal component
PHUs: Primary Health Units
PMAQ-AB: National Program for Improving Access and Quality of Primary Care (*Programa Nacional de Melhoria do Acesso e da Qualidade da Atenção Básica*)
