# Supplementary material for: Evaluation of the primary care for chronic diseases in the high coverage context of the Family Health Strategy
Source: BMC Health Serv Res. 2019 Nov 29;19:913. doi: 10.1186/s12913-019-4737-2 (PMC6884915; doi:10.1186/s12913-019-4737-2)
Supplement: Supplementary file 1 — Additional file 1. Description of the variables of the PHUs (n = 233) and the family health teams (n = 266). [file 12913_2019_4737_MOESM1_ESM.docx]

**Additional file 1:** Description of the variables of the PHUs (n=233) and the family health teams (n=266).

| **Variables** | **Cycle 1** | | | **Cycle 2** | | |
| --- | --- | --- | --- | --- | --- | --- |
|  | **n** | **%(95%CI)** | **m.v** | **n** | **%(95%CI)** | **m.v** |
| **Structure of the PHUs** |  |  |  |  |  |  |
| Complete minimum team | 226 | 97.0 (93.9–98.5) | 0 | 231 | 99.1 (96.9–99.8) | 0 |
| Open at lunch time | 115 | 49.4 (43.0–55.7) | 0 | 78 | 33.5 (27.7–39.8) | 0 |
| Open in the morning and afternoon. | 231 | 99.1 (96.9–99.8) | 0 | 232 | 99.6 (97.6–99.9) | 0 |
| Open 5 days a week | 217 | 93.1 (92.1–97.6) | 6 | 229 | 98.3 (95.7–99.3) | 0 |
| Open over the weekend | 10 | 4.3 (2.4–7.9) | 6 | 17 | 7.3 (4.6–11.4) | 0 |
| Reception and waiting room | 208 | 89.3 (84.6–92.6) | 0 | 230 | 98.7 (96.3–99.6) | 0 |
| Room for collective activities | 81 | 34.8 (28.9–41.1) | 0 | 104 | 44.6 (38.4–51.1) | 0 |
| Procedures room | 111 | 47.6 (41.3–54.0) | 0 | 204 | 87.6 (82.7–91.2) | 0 |
| Observation room | 51 | 21.9 (17.1–27.6) | 0 | 140 | 60.1 (53.7–66.2) | 0 |
| Reception room | 77 | 33.0 (27.5–39.5) | 0 | 154 | 66.1 (59.8–71.9) | 0 |
| Inhalation room | 33 | 14.2 (10.3–19.2) | 0 | 177 | 76.0 (70.1–81.0) | 0 |
| Dressings room | 168 | 72.1 (66.0–77.5) | 0 | 210 | 90.1 (85.6–93.3) | 0 |
| Utility room | 66 | 28.3 (22.9–34.4) | 0 | 79 | 33.9 (28.1–40.2) | 0 |
| Sterilization room | 114 | 48.9 (42.6–55.3) | 0 | 166 | 71.2 (65.1–76.7) | 0 |
| Vaccination room | 212 | 91.0 (86.6–94.0) | 0 | 208 | 89.3 (84.6–92.6) | 0 |
| Clinical consulting room | 232 | 99.6 (97.6–99.9) | 0 | 226 | 97.0 (94.5–98.8) | 1 |
| Clinical examination table | 229 | 98.3 (95.7–99.3) | 0 | 229 | 98.3 (95.7–99.3) | 0 |
| Anthropometric scale up to 200 kg | 215 | 92.3 (88.1–95.1) | 0 | 215 | 92.3 (88.1–95.1) | 0 |
| Sphygmomanometer and stethoscope | 215 | 92.3 (88.1–95.1) | 0 | 221 | 94.8 (91.2–97.0) | 0 |
| Glucose meter and reagent strips | 166 | 71.2 (65.1–76.7) | 0 | 197 | 84.5 (79.4–88.6) | 0 |
| Esthesiometer | 173 | 74.2 (68.6–79.7) | 1 | 176 | 75.5 (69.6–80.6) | 0 |
| Wheelchair | 77 | 33.0 (27.3–39.3) | 0 | 112 | 48.1 (41.7–54.5) | 0 |
| Computer | 149 | 63.9 (57.6–69.8) | 0 | 164 | 70.4 (64.2–75.9) | 0 |
| Team with internet access | 104 | 44.6 (38.4–51.1) | 0 | 108 | 46.4 (40.1–52.8) | 0 |
| Team with Telehealth | 46 | 19.7 (15.1–25.3) | 0 | 46 | 19.7 (15.1–25.3) | 0 |
| Vehicle for external activities | 140 | 60.1 (53.7–66.2) | 0 | 128 | 54.9 (48.5–61.2) | 0 |
| Speculum | 221 | 94.8 (91.2–97.0) | 0 | 212 | 91.0 (86.6–94.0) | 0 |
| Endocervical brush | 221 | 94.8 (91.2–97.0) | 0 | 207 | 88.8 (84.2–92.3) | 0 |
| Ayre spatula | 221 | 94.8 (91.2–97.0) | 0 | 208 | 89.3 (84.6–92.6) | 0 |
| Slide clamp | 222 | 95.3 (91.8–97.3) | 0 | 209 | 89.7 (85.1–93.0) | 0 |
| Glass blade with matte side | 220 | 94.4 (90.7–96.7) | 0 | 211 | 90.6 (86.1–93.7) | 0 |
| Blade holder | 201 | 86.3 (81.3–90.1) | 0 | 204 | 87.6 (82.7–91.2) | 0 |
| Light focuser | 220 | 94.4 (90.7–96.7) | 0 | 224 | 96.1 (92.8–98.0) | 0 |
| Gynecological table | 222 | 95.3 (91.8–97.3) | 0 | 225 | 96.6 (93.4–98.3) | 0 |
| Prednisone | 102 | 43.8 (37.6–50.2) | 0 | 58 | 24.9 (19.8–30.8) | 0 |
| Salbutamol | 81 | 34.8 (28.9–41.1) | 0 | 78 | 33.5 (27.7–39.8) | 0 |
| Ipratropium bromide | 83 | 35.6 (29.8–42.0) | 0 | 76 | 32.6 (26.9–38.9) | 0 |
| Beclomethasone | 22 | 9.4 (6.3–13.9) | 0 | 31 | 13.3 (9.5–18.3) | 0 |
| Fenoterol | 87 | 37.3 (31.4–43.7) | 0 | 20 | 8.6 (5.6–12.9) | 0 |
| Captopril | 39 | 16.7 (12.5–22.1) | 0 | 102 | 43.8 (37.6–50.2) | 0 |
| Beta blockers | 125 | 53.6 (47.2–59.9) | 0 | 110 | 47.2 (40.9–53.6) | 0 |
| Hydrochlorothiazide | 122 | 52.4 (46.0–58.7) | 0 | 93 | 39.9 (33.8–46.3) | 0 |
| Losartan | 69 | 29.6 (24.1–35.8) | 0 | 76 | 32.6 (26.9–38.9) | 0 |
| Verapamil | 11 | 4.7 (2.7–8.3) | 0 | 11 | 4.7 (2.7–8.3) | 0 |
| Simvastatin | 55 | 23.6 (18.6–29.5) | 0 | 56 | 24.0 (19.0–29.9) | 0 |
| Glibenclamide | 126 | 54.1 (47.7–60.4) | 0 | 100 | 42.9 (36.7–49.3) | 0 |
| Metformin | 124 | 53.2 (46.8–59.5) | 0 | 101 | 43.3 (37.1–49.8) | 0 |
| NPH Insulin | 90 | 38.6 (32.6–45.0) | 0 | 92 | 39.5 (33.4–45.9) | 0 |
| Regular Insulin | 82 | 35.2 (29.4–41.5) | 0 | 83 | 35.6 (29.8–42.0) | 0 |
| Seasonal influenza vaccine | 116 | 49.8 (43.4–56.2) | 0 | 197 | 84.5 (79.4–88.6) | 0 |
| Pneumococcal polysaccharide vaccine | 74 | 31.8 (26.1–38.0) | 0 | 41 | 17.6 (13.2–23.0) | 0 |
| Provide reception | 168 | 72.1 (66.0–77.5) | 0 | 223 | 95.7 (92.3–97.7) | 0 |
| Provide vaccination | 220 | 94.4 (90.7–96.7) | 0 | 216 | 92.7 (88.6–95.4) | 0 |
| Provide medical consultations | 226 | 97.0 (93.9–98.5) | 0 | 230 | 98.7 (96.3–99.6) | 0 |
| Provide nursing consultations | 233 | 100 (98.4–100) | 0 | 231 | 99.1 (96.9–99.8) | 0 |
| **Work process of the teams** |  |  |  |  |  |  |
| Health education schedule | 205 | 77.1 (71.7–81.7) | 0 | 239 | 89.8 (85.6–92.9) | 0 |
| Health education covering the use of medicinal plants and herbal medicines | 6 | 2.3 (1.0–4.8) | 0 | 13 | 4.9 (2.9–8.2) | 0 |
| Health education for women | 248 | 93.2 (89.6–95.7) | 0 | 222 | 83.5 (78.5–87.4) | 0 |
| Health education for older adults | 245 | 92.1 (88.2–94.8) | 0 | 223 | 83.8 (78.9–87.8) | 0 |
| Health education addressing healthy eating | 232 | 87.2 (82.7–90.7) | 0 | 203 | 76.3 (70.9–81.0) | 0 |
| Health education for men | 118 | 44.4 (38.5–50.4) | 0 | 179 | 67.3 (61.4–72.3) | 0 |
| Health education for the prevention of alcohol and other drugs | 69 | 25.9 (21.0–31.5) | 0 | 104 | 39,1 (34.2–46.1) | 6 |
| Document that proves the performance of health education | 218 | 82.0 (76.9–86.1) | 0 | 198 | 74.4 (68.9–79.0) | 0 |
| Physical activity | 136 | 51.1 (45.2–57.1) | 0 | 187 | 70.3 (64.6–75.5) | 0 |
| Activities in schools | 171 | 64.3 (58.4–69.8) | 0 | 186 | 69.9 (64.2–75.1) | 0 |
| Registration of schoolchildren with health needs | 62 | 23.3 (18.6–28.8) | 0 | 92 | 34.6 (29.1–40.5) | 0 |
| Clinical evaluation of school children for NCDs | 109 | 41.0 (35.2–47.0) | 0 | 135 | 50.8 (44.8–56.7) | 0 |
| Provide integrative and complementary practices | 19 | 7.1 (4.6–10.9) | 0 | 32 | 12.0 (8.7–16.5) | 0 |
| Evaluation of user satisfaction | 75 | 28.2 (23.1–33.9) | 0 | 143 | 53.8 (47.8–59.7) | 0 |
| Channels of communication with users | 186 | 69.9 (64.2–75.1) | 0 | 166 | 62.4 (56.5–68.0) | 0 |
| Consider the opinions of the users in the planning | 218 | 82.0 (76.9–86.1) | 0 | 244 | 91.7 (87.8–94.5) | 0 |
| Provide actions for women’s groups (cancer prevention) | 237 | 89.1 (84.8–92.3) | 0 | 230 | 86.5 (81.8–90.1) | 0 |
| Provide actions for groups of people with obesity | 116 | 43.6 (37.8–49.6) | 0 | 123 | 46.2 (40.4–52.2) | 0 |
| Provide actions for groups of people with hypertension | 252 | 94.7 (91.4–96.8) | 0 | 245 | 92.1 (88.2–94.8) | 0 |
| Provide actions for groups of people with diabetes | 251 | 94.4 (90.9–96.6) | 0 | 244 | 91.7 (87.8–94.5) | 0 |
| Provide actions for groups of people with chronic lung disease | 107 | 40.2 (36.2–48.3) | 12 | 93 | 35.0 (29.5–40.9) | 0 |
| Receptive to spontaneous requests | 157 | 59.0 (53.0–64.8) | 0 | 259 | 97.4 (94.7–98.7) | 0 |
| Service for moving users | 216 | 81.2 (76.1–85.4) | 0 | 241 | 90.6 (86.5–93.6) | 0 |
| Waiting time of up to 1 hour in the reception | 264 | 99.2 (97.3–99.8) | 0 | 263 | 98.9 (96.7–99.6) | 0 |
| Scheduling on any day of the week and at any time | 117 | 44.0 (38.2–50.0) | 0 | 152 | 57.1 (51.1–63.0) | 0 |
| Schedule to provide continued care | 254 | 95.5 (93.2–97.9) | 2 | 246 | 92.5 (88.7–95.1) | 0 |
| Provide actions for groups of self-management support for NCDs | 169 | 63.5 (57.6–69.1) | 0 | 169 | 63.5 (57.6–69.1) | 0 |
| Renew prescriptions without marking medical consultation | 215 | 80.8 (76.0–85.4) | 1 | 232 | 87.2 (82.7–90.7) | 0 |
| Protocol for priority home visits | 100 | 37.6 (32.0–43.6) | 0 | 98 | 36.8 (31.3–42.8) | 0 |
| Home visits schedule | 234 | 88.0 (94.7–98.9) | 26 | 259 | 97.4 (94.7–98.7) | 0 |
| Home visits according to risk and vulnerability assessment | 249 | 93.6 (90.4–96.3) | 1 | 259 | 97.4 (94.7–98.7) | 0 |
| Community health workers carry out priority visits | 246 | 92.5 (89.1–95.4) | 1 | 255 | 95.9 (92.8–97.7) | 0 |
| Clinical care for housebound or bedridden people | 262 | 98.5 (96.7–99.6) | 1 | 256 | 96.2 (93.2–98.0) | 0 |
| Registration of bedridden people | 117 | 44.0 (38.2–50.0) | 0 | 132 | 49.6 (43.7–55.6) | 0 |
| Management provides information for health situation analysis | 240 | 90.2 (86.1–93.2) | 0 | 249 | 93.6 (90.0–96.0) | 0 |
| Self-assessment in the last 6 months | 220 | 82.7 (79.4–88.2) | 5 | 210 | 78.9 (76.5–86.0) | 9 |
| Self-assessment with the AMAQ | 186 | 69.9 (64.2–75.1) | 0 | 167 | 62.8 (56.8–68.4) | 0 |
| Team meeting | 256 | 96.2 (94.1–98.5) | 2 | 264 | 99.2 (97.3–99.8) | 0 |
| Risk and vulnerability criteria for ascribed population | 130 | 48.9 (44.9–57.1) | 11 | 179 | 67.3 (68.4–79.4) | 25 |
| Maps with the area marked | 192 | 72.2 (66.5–77.2) | 0 | 216 | 81.2 (76.1–85.4) | 0 |
| Records organized by family nuclei | 93 | 35.0 (29.5–40.9) | 0 | 104 | 39.1 (33.4–45.1) | 0 |
| Implemented electronic medical charts | 20 | 7.5 (4.9–11.3) | 0 | 32 | 12.0 (8.7–16.5) | 0 |
| Local health council and spaces of public participation | 123 | 46.2 (40.4–52.2) | 0 | 84 | 31.6 (26.3–37.4) | 0 |
| Monitoring and analysis of indicators and health information | 221 | 83.1 (79.5–88.3) | 4 | 248 | 93.2 (89.6–95.7) | 0 |
| Matrix support in the resolution of complex cases | 148 | 55.6 (49.8–61.7) | 1 | 245 | 92.1 (88.6–95.1) | 1 |
| Specialized consultation immediately scheduled by the PHUs | 38 | 14.3 (10.6–19.0) | 0 | 52 | 19.5 (15.2–24.7) | 0 |
| Specialized consultation scheduled later by the PHUs | 160 | 60.2 (54.2–65.9) | 0 | 138 | 51.9 (45.9–57.8) | 0 |
| Specialized consultation scheduled by the user in the booking central | 53 | 19.9 (15.6–25.1) | 0 | 74 | 27.8 (22.8–33.5) | 0 |
| User receives referral form to seek scheduling | 113 | 42.5 (36.7–48.5) | 0 | 142 | 53.4 (47.4–59.3) | 0 |

Definitions of abbreviations: PHUs = primary health units; m.v = missing value; NPH = Neutral Protamine Hagedorn; NCDs = chronic noncommunicable diseases; AMAQ = self-assessment for quality improvement (*autoavaliação para melhoria da qualidade*) instrument.
