# Supplementary material for: Evaluation of the primary care for chronic diseases in the high coverage context of the Family Health Strategy
Source: BMC Health Serv Res. 2019 Nov 29;19:913. doi: 10.1186/s12913-019-4737-2 (PMC6884915; doi:10.1186/s12913-019-4737-2)
Supplement: Supplementary file 2 — Additional file 2. Factor loading of the variables of the PHUs structure. [file 12913_2019_4737_MOESM2_ESM.docx]

**Additional file 2:** Factor loading of the variables of the PHUs structure.

| **Variables** | **Cycle 1** | | | **Cycle 2** | | |
| --- | --- | --- | --- | --- | --- | --- |
|  | **Principal Components** | | | **Principal Components** | | |
|  | **1** | **2** | **3** | **1** | **2** | **3** |
| Seasonal influenza vaccine | 0.139 | 0.142 | **0.400^†^** | 0.197 | 0.251 | **0.519^†^** |
| Pneumococcal polysaccharide vaccine | 0.008 | 0.144 | 0.311 | 0.092 | 0.145 | 0.217 |
| Provide reception | 0.014 | 0.051 | 0.204 | 0.002 | 0.251 | 0.013 |
| Provide vaccination | 0.166 | 0.077 | **0.416^†^** | 0.106 | 0.300 | **0.522^†^** |
| Provide medical consultations | 0.066 | 0.062 | 0.300 | 0.072 | 0.349 | 0.000 |
| Provide nursing consultations | * | * | * | 0.145 | **0.401^†^** | 0.018 |
| Gynecological table | 0.043 | **0.515^†^** | 0.028 | 0.057 | 0.066 | 0.077 |
| Prednisone | **0.797^†^** | 0.118 | 0.044 | **0.638^†^** | 0.057 | 0.130 |
| Salbutamol | **0.679^†^** | 0.059 | 0.166 | **0.807^†^** | 0.005 | 0.118 |
| Ipratropium bromide | **0.678^†^** | 0.016 | 0.007 | **0.804^†^** | 0.109 | 0.013 |
| Beclomethasone | 0.338 | 0.047 | 0.142 | **0.469^†^** | 0.197 | 0.038 |
| Fenoterol | **0.714^†^** | 0.018 | 0.050 | 0.291 | 0.153 | 0.073 |
| Captopril | **0.439^†^** | 0.022 | 0.020 | **0.900^†^** | 0.180 | 0.099 |
| Beta blockers | **0.816^†^** | 0.067 | 0.205 | **0.936^†^** | 0.157 | 0.063 |
| Hydrochlorothiazide | **0.829^†^** | 0.115 | 0.191 | **0.873^†^** | 0.192 | 0.072 |
| Losartan | **0.646^†^** | 0.130 | 0.040 | **0.778^†^** | 0.215 | 0.069 |
| Verapamil | 0.271 | 0.116 | 0.177 | 0.311 | 0.056 | 0.065 |
| Simvastatin | **0.545^†^** | 0.070 | 0.192 | **0.695^†^** | 0.026 | 0.153 |
| Glibenclamide | **0.881^†^** | 0.120 | 0.217 | **0.876^†^** | 0.172 | 0.103 |
| Metformin | **0.830^†^** | 0.126 | 0.215 | **0.895^†^** | 0.153 | 0.104 |
| NPH Insulin | **0.771^†^** | 0.036 | 0.001 | **0.864^†^** | 0.140 | 0.122 |
| Regular Insulin | **0.735^†^** | 0.064 | 0.058 | **0.814^†^** | 0.135 | 0.114 |
| Complete minimum team | 0.076 | 0.016 | 0.299 | 0.100 | 0.008 | 0.044 |
| Open at lunch time | 0.052 | 0.146 | 0.348 | 0.037 | 0.294 | 0.164 |
| Open in the morning and afternoon | 0.065 | 0.033 | 0.068 | 0.060 | 0.214 | 0.087 |
| Open 5 days a week | 0.202 | 0.082 | 0.061 | 0.078 | 0.056 | 0.059 |
| Open over the weekend | 0.202 | 0.082 | 0.061 | 0.331 | 0.075 | 0.074 |
| Reception and waiting room | 0.002 | 0.074 | 0.174 | 0.044 | 0.049 | 0.234 |
| Room for collective activities | 0.240 | 0.166 | 0.298 | 0.064 | 0.134 | 0.354 |
| Procedures room | 0.112 | 0.028 | 0.146 | 0.041 | 0.137 | **0.509^†^** |
| Observation room | 0.398 | 0.100 | 0.244 | 0.304 | 0.169 | 0.302 |
| Reception room | 0.032 | 0.186 | 0.300 | 0.001 | 0.267 | 0.212 |
| Inhalation room | 0.154 | 0.150 | 0.036 | 0.191 | 0.049 | **0.430^†^** |
| Dressings room | 0.006 | 0.198 | 0.227 | 0.080 | 0.091 | **0.477^†^** |
| Utility room | 0.191 | 0.123 | 0.346 | 0.092 | 0.059 | 0.198 |
| Sterilization room | 0.153 | 0.130 | **0.559^†^** | 0.085 | 0.206 | **0.429^†^** |
| Vaccination room | 0.183 | 0.198 | **0.477^†^** | 0.195 | 0.312 | **0.534^†^** |
| Clinical consulting room | 0.100 | 0.245 | 0.107 | 0.029 | 0.037 | 0.059 |
| Clinical examination table | 0.075 | 0.056 | 0.056 | * | * | * |
| Anthropometric scale up to 200kg | 0.237 | 0.185 | 0.016 | 0.161 | 0.012 | 0.269 |
| Sphygmomanometer and stethoscope | 0.069 | 0.009 | 0.197 | 0.091 | 0.040 | 0.091 |
| Glucose meter and reagent strips | 0.308 | 0.270 | 0.167 | 0.137 | **0.398** | 0.157 |
| Computer | 0.328 | 0.151 | 0.363 | 0.265 | 0.361 | 0.071 |
| Team with internet access | 0.349 | 0.085 | **0.433^†^** | 0.055 | 0.274 | **0.418^†^** |
| Team with Telehealth | 0.338 | 0.168 | 0.188 | 0.227 | 0.224 | 0.091 |
| Wheelchair | 0.320 | 0.096 | 0.214 | 0.291 | 0.037 | 0.240 |
| Vehicle for external activities | 0.170 | 0.054 | **0.525^†^** | 0.133 | 0.132 | **0.422^†^** |
| Speculum | 0.062 | **0.711^†^** | 0.000 | 0.084 | **0.878^†^** | 0.234 |
| Endocervical brush | 0.041 | **0.770^†^** | 0.305 | 0.145 | **0.844^†^** | 0.235 |
| Ayre spatula | 0.037 | **0.816^†^** | 0.236 | 0.075 | **0.813^†^** | 0.258 |
| Slide clamp | 0.006 | **0.715^†^** | 0.195 | 0.150 | **0.718^†^** | 0.167 |
| Glass blade with matte side | 0.094 | **0.653^†^** | 0.284 | 0.186 | **0.692^†^** | 0.146 |
| Blade holder | 0.048 | **0.463^†^** | 0.357 | 0.129 | **0.765^†^** | 0.320 |
| Light focuser | 0.034 | **0.435^†^** | 0.018 | 0.106 | 0.061 | 0.046 |
| Esthesiometer | 0.095 | 0.370 | 0.267 | 0.104 | 0.166 | 0.234 |
| **Eigenvalue** | 8.59 | 4.16 | 2.96 | 19.54 | 10.95 | 5.66 |
| **Variance (%)** | 15.91 | 7.70 | 5.30 | 18.26 | 10.23 | 5.30 |
| **Cronbach’s alpha** | 0.900 | 0.774 | 0.674 | 0.958 | 0.917 | 0.831 |
| **Scores, median (IQR)** | 0,050 (-0,820; 0,970) | -0,230 (-0,530; 0,080) | -0,120 (-0,790; 0,760) | -0,485 (-0,890; 0,880) | 0,290 (-0,130; 0,520) | 0,080 (-0,430; 0,570) |

Definitions of abbreviations: PHUs = primary health units; NPH = Neutral Protamine Hagedorn; IQR = Interquartile Range. PC1: Medications for the treatment of NCDs; PC2: materials for cervical cancer screening; PC3: Provision of vaccinations and infrastructure.

Definitions of symbols: †= Factor loading value ≥0.4; *= Values without variation.
