# Supplementary material for: Evaluation of the primary care for chronic diseases in the high coverage context of the Family Health Strategy
Source: BMC Health Serv Res. 2019 Nov 29;19:913. doi: 10.1186/s12913-019-4737-2 (PMC6884915; doi:10.1186/s12913-019-4737-2)
Supplement: Supplementary file 3 — Additional file 3. Factor loading of the variables of the work process of family health teams. [file 12913_2019_4737_MOESM3_ESM.docx]

**Additional file 3:** Factor loading of the variables of the work process of family health teams.

| **Variables** | **Cycle 1** | | **Cycle 2** | |
| --- | --- | --- | --- | --- |
|  | **Principal Components** | | **Principal Components** | |
|  | **1** | **2** | **1** | **2** |
| Health education schedule | 0.374 | -0.244 | **0.400**† | -0.155 |
| Health education covering the use of medicinal plants and herbal medicines | 0.104 | -0.254 | 0.148 | -0.295 |
| Health education for women | **0.532**† | 0.343 | 0.154 | -0.234 |
| Health education for older adults | **0.595**† | 0.338 | 0.285 | -0.355 |
| Health education addressing healthy eating | **0.561**† | 0.243 | 0.288 | -**0.407**† |
| Health education for men | **0.472**† | -0.166 | 0.267 | -0.393 |
| Health education for the prevention of alcohol and other drugs | 0.377 | -0.206 | -0.277 | 0.248 |
| Document that proves the performance of health education | **0.626†** | **0.431†** | 0.370 | -0.191 |
| Physical activities | 0.336† | -0.086 | **0.414**† | -0.119 |
| Activities in schools | 0.346 | 0.012 | 0.379 | -0.119 |
| Registration of schoolchildren with health needs | **0.412**† | -0.236 | **0.410**† | -0.255 |
| Clinical evaluation of school children for NCDs | 0.191 | 0.206 | 0.337 | -0.046 |
| Provide integrative and complementary practices | 0.077 | -0.326 | 0.231 | -0.307 |
| Evaluation of user satisfaction | **0.410**† | -0.137 | **0.465**† | -0.191 |
| Channels of communication with users | 0.207 | -0.205 | 0.132 | -0.327 |
| Consider the opinions of the users in the planning | 0.300 | -0.187 | 0.339 | 0.001 |
| Provide actions for women’s groups (cancer prevention) | 0.266 | -0.225 | **0.464**† | **0.508**† |
| Provide actions for groups of people with obesity | 0.378† | 0.111 | **0.433**† | 0.309 |
| Provide actions for groups of people with hypertension | **0.484**† | **0.521**† | **0.548**† | **0.596**† |
| Provide actions for groups of people with diabetes | **0.453**† | **0.459**† | **0.530**† | **0.596**† |
| Provide actions for groups of people with chronic lung disease | 0.365 | -0.028 | 0.357 | 0.251 |
| Receptive to spontaneous requests | 0.336 | -0.158 | 0.110 | 0.050 |
| Service for removal of users | 0.099 | 0.179 | -0.165 | 0.003 |
| Waiting time of up to 1 hour in the reception | -0.128 | 0.110 | 0.222 | 0.024 |
| Scheduling on any day of the week and at any time | 0.177 | -0.086 | 0.329 | 0.223 |
| Schedule to provide continued care | 0.154 | -0.362 | 0.097 | -0.105 |
| Provide actions for groups of self-management support for NCDs | **0.502**† | -0.132 | 0.323 | -0.337 |
| Renew prescriptions without marking medical consultation | 0.039 | -0.267 | 0.270 | -0.007 |
| Protocol for priority home visits | 0.308 | -0.326 | **0.431**† | 0.022 |
| Home visits schedule | 0.108 | 0.013 | 0.099 | -0.044 |
| Home visits according to risk and vulnerability assessment | 0.231 | -0.059 | 0.289 | 0.128 |
| Community health workers carry out priority visits | 0.214 | -0.009 | 0.322 | 0.151 |
| Clinical care for housebound or bedridden people | -0.063 | 0.070 | 0.267 | 0.040 |
| Registration of bedridden people | **0.416**† | -0.357 | **0.531**† | -0.201 |
| Management provides information for health situation analysis | 0.335 | -0.023 | **0.446**† | 0.112 |
| Self-assessment in the last 6 months | 0.347 | -0.197 | 0.280 | -0.230 |
| Self-assessment with the AMAQ | 0.345 | 0.040 | 0.323 | -0.227 |
| Staff meeting | 0.302 | 0.224 | 0.084 | -0.041 |
| Risk and vulnerability criteria for ascribed population | 0.206 | -0.256 | **0.474**† | 0.178 |
| Maps with the area marked | 0.377 | -0.107 | 0.392 | -0.118 |
| Records organized by family nuclei | 0.105 | 0.134 | -0.104 | -0.287 |
| Implemented electronic medical charts | 0.160 | -0.333 | 0.325 | -0.288 |
| Local health council and spaces of public participation | 0.141 | 0.246 | 0.190 | 0.115 |
| Monitoring and analysis of indicators and health information | 0.333 | -0.227 | 0.363 | 0.121 |
| Matrix support in the resolution of complex cases | 0.066 | -0.173 | 0.214 | -0.135 |
| Specialized consultation immediately scheduled by the PHUs | 0.017 | -0.140 | 0.189 | 0.204 |
| Specialized consultation scheduled later by the PHUs | 0.144 | -0.274 | 0.205 | -0.181 |
| Specialized consultation scheduled by the user in the booking central | -0.056 | 0.279 | -0.006 | 0.312 |
| User receives referral form to seek scheduling | -0.068 | 0.104 | 0.104 | **0.409**† |
| **Eigenvalue** | 5.05 | 2.69 | 5.07 | 3.15 |
| **Variance (%)** | 10.03 | 5.49 | 11.16 | 6.42 |
| **Cronbach’s alpha** | 0.819 | 0.642 | 0.819 | 0.696 |
| **Scores, median (IQR)** | -0,165 (-0,627; 0,437) | -0,066 (-0,543; 0,494) | 0,171 (-0,593; 1,124) | 0,063 (-0,507; 1,216) |

Definitions of abbreviations: NCDs = chronic noncommunicable diseases; AMAQ = self-assessment for quality improvement (*autoavaliação para melhoria da qualidade*) instrument; IQR = Interquartile Range; PHUs = primary health units.

Cycle 1: PC1: Health Promotion; PC2: Care for groups with diabetes and hypertension; Cycle 2: PC1: Health promotion and health site analysis; PC2: Health education and user referral

Definitions of symbols: † = Factor loading value ≥ 0.4
