# Supplementary figures and images for: Evaluation of the primary care for chronic diseases in the high coverage context of the Family Health Strategy
Source: BMC Health Serv Res. 2019 Nov 29;19:913. doi: 10.1186/s12913-019-4737-2 (PMC6884915; doi:10.1186/s12913-019-4737-2)

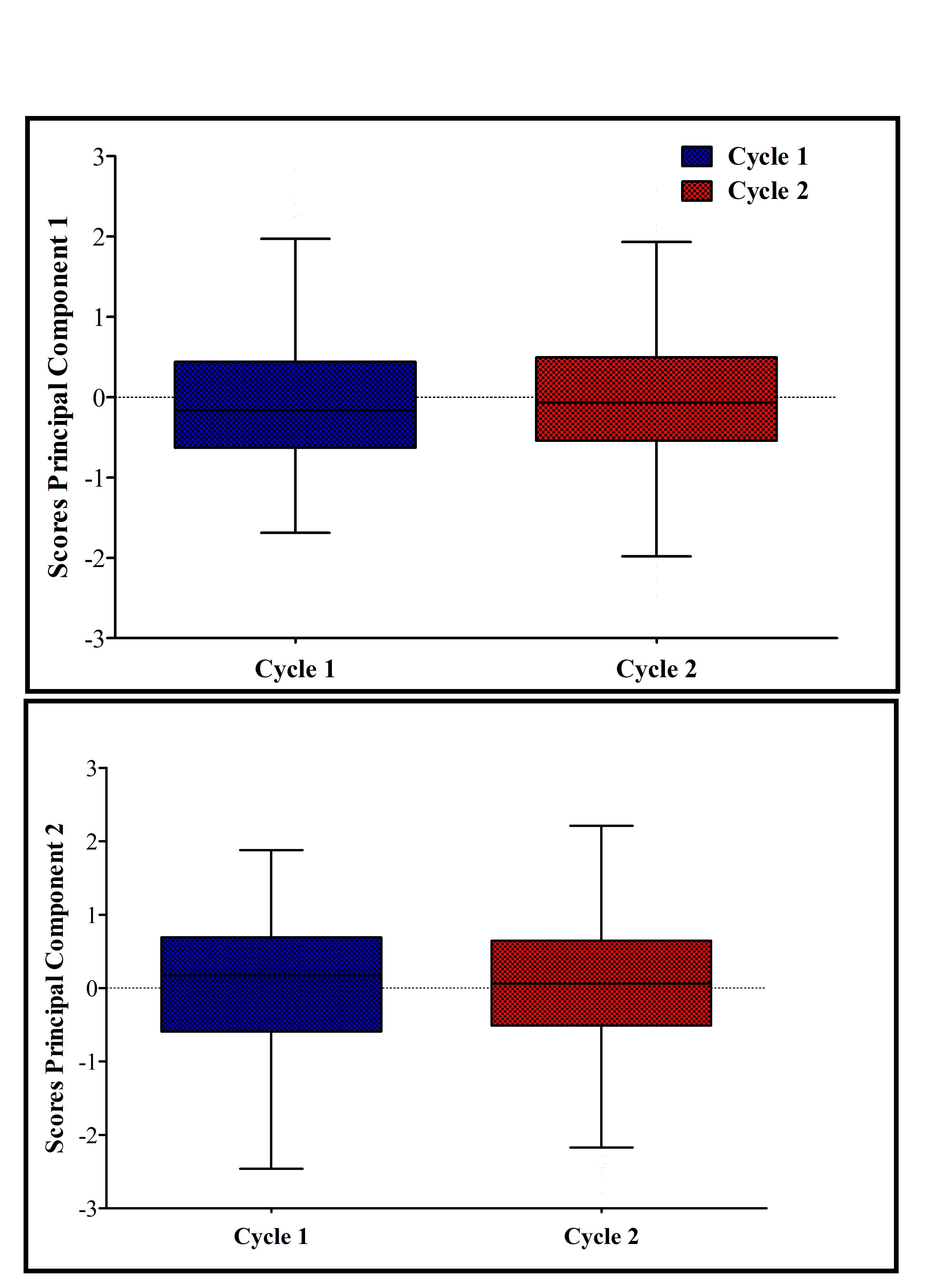

Supplement: Supplementary file 5 — Additional file 5: Figure S2. Descriptive analysis of principal component scores between Cycles 1 and 2 for family health teams. Definitions of abbreviations: Cycle 1 - PC1: Health Promotion; PC2: Care for groups with diabetes and hypertension; Cycle 2 - PC1: Health promotion and health site analysis; PC2: Health education and user referral. [file 12913_2019_4737_MOESM5_ESM.tif]
